# Neurodiversity and mental health in adulthood: exploring the unique contributions of autism and ADHD to internalising problems

**DOI:** 10.1038/s41598-026-35440-6

**Published:** 2026-04-01

**Authors:** Luca D. Hargitai, Lucy H. Waldren, Lucy A. Livingston, Florence Y. N. Leung, Punit Shah

**Affiliations:** 1 Department of Psychology, University of Bath, Bath, UK; 2 Department of Psychology, Institute of Psychiatry, Psychology and Neuroscience, King’s College London, London, UK; 3 School of Social Sciences, College of Health, Science and Society, University of the West of England, Bristol, UK

**Keywords:** Autism, ADHD, Neurodevelopmental conditions, Anxiety, Depression, Internalising problems, Psychology, Human behaviour

## Abstract

Autism and Attention Deficit Hyperactivity Disorder (ADHD) are both linked to internalising problems (e.g., anxiety, depression) but their frequent co-occurrence makes these relationships difficult to disentangle. We, therefore, adopted a trait-based approach to examine the unique associations of autism and ADHD with internalising problem diagnoses in a large general population sample of adults from the United Kingdom and United States (*N* = 4996). We then assessed whether these associations would conceptually replicate in a clinical sub-sample (*n* = 292) using a case-control design. We found that, across the whole sample, ADHD traits were an overall stronger predictor of internalising problem diagnoses than autistic traits. The case-control analyses revealed that both autistic adults and adults with ADHD had greater odds of being diagnosed with anxiety, depression and an overall internalising problem (i.e., depression and/or anxiety) than neurotypical adults. Although the clinical groups did not significantly differ from each other in their associations with mental health diagnoses, having and ADHD diagnosis was more closely linked with depression while having an autism diagnosis was more closely linked with anxiety. We discuss potential psychological mechanisms underlying these findings and the implications for research and clinical practice concerning neurodevelopmental conditions.

**Supplementary Information:**

The online version contains supplementary material available at 10.1038/s41598-026-35440-6.

## Introduction

Autism spectrum disorder (hereafter autism) is a lifelong neurodevelopmental condition characterised by social communication differences, focused interests and varying sensory preferences^1^. Although there is increasing recognition of psychological strengths that may be features of autism^2,3^, recent research has highlighted that autistic individuals often experience difficulties in various domains, including relationships, education, work, and overall quality of life (e.g., ^4–6^), which some have linked to a societal failure to accommodate the physical, social and emotional needs of autistic people (e.g., ^7,8^). There is also increasing evidence of an association between autism and mental health outcomes in adulthood. For example, autism has been statistically linked to schizophrenia, various mood disorders, a range of anxiety disorders and personality disorders (see^9^). Taking a closer look at internalising problems, Hollocks and colleagues^10^ recently showed that depression and anxiety are among the most frequently experienced psychiatric conditions in autistic adults, with prevalence rates estimated to be as high as 37% and 42%, respectively. This leads to further reductions in quality of life, creating additional barriers for autistic adults^6,11^.

Attention-deficit hyperactivity disorder (ADHD) – characterised by inattention and/or hyperactivity and impulsivity^1^ – is another neurodevelopmental condition that has been linked to poor life outcomes^12,13^, despite a growing awareness of its potential positive aspects^14,15^. Although historically thought of as a childhood disorder^16^, ADHD is estimated to occur in approximately 3% of the adult population^17^ and is associated with employment-related difficulties, relationship breakdowns, and a lower quality of life^16,18,19^. Mental health problems are also more prevalent among adults with ADHD than in the general population, with a recent meta-analysis indicating that internalising problems, like anxiety and depression, are among the most common psychiatric comorbidities^20^. Notably, individuals with internalising problems with co-occurring ADHD have poorer overall outcomes than those without ADHD^21,22^. At the same time, co-occurring anxiety and depression is associated with greater social difficulties in people with ADHD^23,24^ and may underpin the link between childhood ADHD and lower quality of life in adulthood^25^.

It is becoming increasingly clear that both autism and ADHD are linked to internalising problems, which then have a further negative impact on overall physical health, mental wellbeing, and quality of life in children and adults^26^. However, with a greater clinical and research emphasis on autism^27–29^, the relative impact of the two conditions on mental health is strikingly unclear. Additional complications arise due to an overlap in their symptom profiles (e.g., attentional and emotional difficulties^30,31^) and frequent diagnostic co-occurrence^32^. These factors make it particularly difficult to examine the unique contributions of these neurodevelopmental conditions to mental health outcomes, which is reflected by the dearth of research on this topic. Furthermore, the recent retraction of several studies comparing the psychiatric comorbidities of children with autism and/or ADHD (e.g., ^33,34^) indicates that even less is known about these relationships than originally thought.

Considering these challenges, we recently adopted a trait-based approach, which is increasingly used in neurodevelopmental research (e.g., ^35–37^), to investigate the unique associations of autism and ADHD with mental health outcomes^38^. In a nationally representative sample of adults from the United Kingdom, we showed that ADHD traits were a stronger and statistically more important predictor of internalising symptoms than autistic traits. However, questions remain about the relationship between neurodevelopmental traits and the clinical diagnosis of internalising problems like generalised anxiety disorder (GAD) and depression. In particular, it is unclear whether the findings observed in our initial study would replicate in samples including people with clinically diagnosed neurodevelopmental conditions. Addressing these outstanding questions, the present study aims to determine whether autism or ADHD is more strongly associated with clinically diagnosed internalising problems. Following our previous study^38^, we hypothesised that both autism and ADHD would be significantly associated with internalising problem diagnoses but expected the link with ADHD to be stronger.

## Methods

### Data

Data were drawn from an existing openly accessible dataset including autism and ADHD measures^37^. The sample consists of 5000 adults from the United Kingdom and United States of America. For the current study, participants who identified as non-binary (*n* = 2) and those whose education level was unclear (*n* = 2) were not included. Therefore, our final sample had 4996 participants (2478 [49.60%] male, 2518 [50.40%] female), aged between 18 and 89 years (*M* = 39.18 years, *SD* = 13.39 years). Ethical clearance was granted by the University of Bath Psychology Research Ethics Committee (PREC 19–025), and all participants gave informed consent at the initial data collection for their anonymised data to be used in future analyses. All procedures performed were in accordance with the ethical standards of the institutional committee and with the 1964 Declaration of Helsinki and its later amendments or comparable ethical standards.

### Measures

#### Demographic information and diagnostic status

Participants provided information on their age (years), sex at birth (male, female, non-binary) and education level between 0 (no qualifications) and 8 (PhD or equivalent) on the International Standard Classification of Education^39^. They also reported whether they had any clinically diagnosed mental health conditions. In total, 1889 participants had at least one condition, including anxiety/GAD (*n* = 1185), depression/any depressive disorder (*n* = 1146), ADHD (*n* = 104) and autism (*n* = 90). These rates are broadly representative of the global prevalence of autism and ADHD but are higher than the general population prevalence rates for anxiety and depressive disorders^40^. For information on all diagnoses, see Table 1.

**Table 1:** Clinical diagnoses within the sample (*N* = 4996).

Condition	*n*	%
Anxiety/generalised anxiety disorder	1185	23.72
Social anxiety disorder	299	5.98
Attention deficit hyperactivity disorder	104	2.08
Autism spectrum disorder	90	1.80
Bipolar disorder	49	0.98
Depression/any depressive disorder	1146	22.94
Any eating disorder	130	2.60
Intellectual disability	8	0.16
Obsessive compulsive disorder	124	2.48
Panic disorder	115	2.30
Any personality disorder	52	1.04
Post-traumatic stress disorder	165	3.30
Schizophrenia/any psychotic disorder	16	0.32
Specific phobia	34	0.68
Any substance related or addiction disorder	60	1.20
Other	15	0.30
None	3107	62.19

Note. The list of psychiatric disorders was created based on the DSM-5^1^ and ICD-10^41^ (for more information, see^37^).

#### Autistic and ADHD traits

The 28-item Short Autism Spectrum Quotient (AQ-Short^42^) assessed participants’ social and non-social autistic traits. Participants responded to items (e.g., *‘I notice patterns in things all the time’*) using a 4-point Likert scale that ranged from 1 (*‘definitely agree’*) to 4 (*‘definitely disagree’*). This generated a total AQ-Short score between 28 (few autistic traits) and 112 (many autistic traits) for each participant. The AQ-Short has been shown to be suitable for use in studies where participant sex is included in the modelling, as it is potentially less biased towards the male autism phenotype than other autism trait measures (see^43,44^). In the present study, the AQ-Short had good internal consistency (*α* = 0.84; *ω* = 0.85), and it has previously been shown to have good construct validity with respect to autism diagnosis^42^.

The Adult ADHD Self-Report Scale (ASRS^45^) assessed participants’ ADHD traits. Its 18 items (e.g., *‘How often do you have problems remembering appointments or obligations?’*) measured the frequency of symptoms related to inattention and hyperactivity/impulsivity on a 5-point Likert scale ranging from 0 (*‘never’*) to 4 (*‘very often’*). This produced total scores between 0 (no ADHD traits) and 72 (many ADHD traits) for each participant. Comparing the ASRS to clinician ratings, Kessler et al.^45^ found a 96.2% classification accuracy for ADHD, supporting the measure’s construct validity. The ASRS had good internal consistency (*α* = 0.91; *ω* = 0.91) in the present study.

### Statistical analyses

Data were analysed in R (version 4.4.2^46^). We first assessed the trait-level associations of autism and ADHD with internalising problem diagnoses using a series of multiple logistic regression analyses. Specifically, we examined the relative links of autistic and ADHD traits with three binary outcome variables whilst accounting for participants’ age, sex and education level: (i) overall internalising problem diagnosis (i.e., having a diagnosis of GAD or depression or both versus no diagnosis), (ii) GAD diagnosis (i.e., having a GAD diagnosis versus no diagnosis), (iii) depression diagnosis (i.e., having a depression diagnosis versus no diagnosis). We used the criterion of Cook’s Distance > 0.25 to identify potential multivariate outliers in the data and visually inspected scatterplots of the predictor variables against the logit of each outcome variable to test the assumption of linearity. The presence of potential multicollinearity in these analyses was assessed using the variance inflation factor (VIF) with VIF < 5 indicating acceptable correspondence among predictor variables.

To better understand the relative contributions of autism and ADHD to internalising problem diagnoses beyond trait-level associations, we conceptually replicated the logistic regressions through a series of classical case-control analyses focusing specifically on participants in our large sample with a clinical diagnosis of autism or ADHD. We used the MatchIt package in R (version 4.7.1^47^) to perform nearest neighbour matching^48,49^, allowing us to create an autism-only group, an ADHD-only group, and a neurotypical control group that were matched as closely as possible on age, sex, and education level (for further detail on the matching process, please see the *Statistical Group Matching* section in the Supplementary Materials). To ensure that we obtained distinct clinical groups, we removed any participants who self-reported concurrent autism and ADHD diagnoses (*n* = 17) from the sample prior to commencing the group matching process. To our knowledge, this is the first time that three-way statistical matching has been used to compare autistic-, ADHD-, and neurotypical adults. As the obtained case-control sample of 292 participants (73 autism-only, 73 ADHD-only, and 146 neurotypical) was sufficient to achieve at least 85% statistical power to detect small-to-medium effects (*w* = 0.20, *α* = 0.05), we undertook a series of chi-square tests of independence to compare the groups.

To evaluate the robustness of these case-control analyses, we conducted additional exploratory comparisons of groups created using the AQ-Short and ASRS screeners. A cut-off score of > 65 for total AQ-Short scores was used to categorise participants as potentially autistic versus non-autistic (see^42^). To screen for probable ADHD, we used the first six items of the ASRS. Following Kessler et al.^45^, ratings of 2 (*‘sometimes’*) or above on items 1–3 and ratings of 3 (*‘often’*) or above on items 4–6 were taken to indicate endorsement of a given symptom. Endorsing at least 4 of the 6 symptoms on the screener was used as a threshold for probable ADHD. In total, 2601 participants met the threshold for probable autism and 1334 met the threshold for probable ADHD. As with the clinical groups, we used the MatchIt R package (version 4.7.1^47^) to statistically match the probable autism, probable ADHD and neurotypical control groups on age, sex and education level (for further details on the matching process, please see the *Statistical Group Matching* section in the Supplementary Materials). To ensure that we obtained distinct clinical groups, any participants who met the screening threshold for both autism and ADHD (*n* = 902) were removed from the data prior to commencing the nearest neighbour matching process. The final sample included 432 participants with probable autism, 432 participants with probable ADHD and 864 neurotypical control participants.

The analysis code, which includes further details of the group matching process, and information about the packages used across our analyses are openly available on the Open Science Framework (see 10.17605/OSF.IO/DZVMG).

## Results

### Trait-level associations between autism, ADHD and mental health diagnoses

Based on our predetermined criteria, there were no multivariate outliers, all relationships were approximately linear and, while some predictors were correlated (see Table 2), multicollinearity was not a concern (all VIF values < 1.18).

**Table 2:** Descriptive statistics and correlations between variables.

Measures	Mean (SD)	1	2	3	4	5	6	7
1. Autistic traits	66.20 (10.64)	–						
2. ADHD traits	29.33 (11.41)	0.34***	–					
3. Age (years)	39.18 (13.39)	-0.02	-0.23***	–				
4. Sex	–	0.09***	-0.12***	0.13***	–			
5. Education level	4.87 (1.91)	-0.05***	-0.03*	-0.05***	0.02	–		
6. Internalising problem diagnosis	–	0.17***	0.26***	-0.06***	-0.19***	-0.08***	–	
7. GAD diagnosis	–	0.18***	0.24***	-0.10***	-0.20***	-0.07***	0.99***	–
8. Depression diagnosis	–	0.15***	0.20***	-0.01	-0.14***	-0.06***	0.99***	0.68***

* *p* < 0.05, ** *p* < 0.01, *** *p* < 0.001. Sex is coded as 0 = Female and 1 = Male. Diagnoses are coded as 0 = No Diagnosis and 1 = Diagnosis.

Logistic regression analyses are presented in Table 3. The first analysis revealed that both autistic traits (*b* = 0.02, *p* < 0.001) and ADHD traits (*b* = 0.04, *p* < 0.001) were unique predictors of an internalising problem diagnosis. More specifically, the odds of having a diagnosis of an internalising problem (i.e., GAD and/or depression) increase by 2% with every unit increase in autistic traits and by 4% with every unit increase in ADHD traits.

**Table 3:** Logistic regression analyses.

Predictor	B	SE(B)	z	*p*	OR [95% CI]	VIF
Internalising problems		Overall model fit: *Χ*^*2*^(4990, *N* = 4996) = 462.69, *p* < 0.001, *McFadden R*^*2*^ = 0.07				
Autistic Traits	0.02	0.00	7.35	< 0.001	1.02 [1.02, 1.03]	1.11
ADHD Traits	0.04	0.00	13.55	< 0.001	1.04 [1.04, 1.05]	1.14
Age	0.00	0.00	0.37	0.715	1.00 [1.00, 1.01]	1.07
Sex	-0.48	0.06	-7.36	< 0.001	0.62 [0.55, 0.71]	1.04
Education Level	-0.08	0.02	-4.64	< 0.001	0.93 [0.90, 0.96]	1.00
GAD		Overall Model Fit: *Χ*^*2*^(4990, *N* = 4996) = 445.57, *p* < 0.001, *McFadden R*^*2*^ = 0.08				
Autistic Traits	0.03	0.00	8.64	< 0.001	1.03 [1.02, 1.04]	1.11
ADHD Traits	0.04	0.00	11.42	< 0.001	1.04 [1.03, 1.05]	1.13
Age	-0.01	0.00	-3.60	< 0.001	0.99 [0.98, 1.00]	1.06
Sex	-0.52	0.07	-7.28	< 0.001	0.59 [0.51, 0.68]	1.04
Education Level	-0.07	0.02	-4.08	< 0.001	0.93 [0.90, 0.96]	1.00
Depression		Overall Model Fit: *Χ*^*2*^(4990, *N* = 4996) = 296.42, *p* < 0.001, *McFadden R*^*2*^ = 0.06				
Autistic Traits	0.02	0.00	6.77	< 0.001	1.02 [1.02, 1.03]	1.12
ADHD Traits	0.04	0.00	10.83	< 0.001	1.04 [1.03, 1.04]	1.17
Age	0.01	0.00	2.81	0.005	1.01 [1.00, 1.01]	1.08
Sex	-0.38	0.07	-5.33	< 0.001	0.68 [0.59, 0.79]	1.04
Education Level	-0.07	0.02	-3.71	< 0.001	0.94 [0.90, 0.97]	1.00

Sex is coded as 0 = Female and 1 = Male.

The subsequent logistic regression analyses allowed us to separately examine the associations of these neurodevelopmental traits with GAD and depression. While both autistic traits (GAD: *b* = 0.03, *p* < 0.001; depression: *b* = 0.02, *p* < 0.001) and ADHD traits (GAD: *b* = 0.04, *p* < 0.001; depression: *b* = 0.04, *p* < 0.001) were predictive of GAD and depression diagnoses, there was a stronger association between ADHD traits and diagnoses. Moreover, with every unit increase in ADHD traits, the odds of having a GAD diagnosis increased by 4% and the odds of having a depression diagnosis also increased by 4%. In comparison, a unit increase in autistic traits only led to a 3% increase in the odds of having a GAD diagnosis and a 2% increase in the odds of having a depression diagnosis.

### Clinical-level associations between autism, ADHD and mental health diagnoses

Group comparisons, shown in Table 4, indicate that the autism-only, ADHD-only and neurotypical control groups were well-matched on age, sex and education level. Chi-square tests of independence were used to compare the odds of having a mental health diagnosis between groups. The first analysis showed a significant association between neurodevelopmental condition diagnosis and internalising problem diagnosis: *Χ*^*2*^(2, *N* = 292) = 37.66, *p* < 0.001, *V* [95% CI] = 0.36 [0.25, 0.48]. Follow-up tests indicated that people in both the autism and ADHD groups were significantly more likely to be diagnosed with an internalising problem than the neurotypical control group (autism: *Χ*^*2*^ = 21.97, *Bonferroni-corrected p* < 0.001; ADHD: *Χ*^*2*^ = 29.48, *Bonferroni-corrected p* < 0.001). Indeed, both the autism-only (*OR* [95% CI] = 3.94 [2.19, 7.22]) and the ADHD-only group (*OR* [95% CI] = 5.00 [2.75, 9.31]) had greater odds of having an internalising problem diagnosis than the neurotypical control group. Interestingly, while the ADHD-only group had a higher odds ratio, it did not significantly differ from the autism-only group in its likelihood of having an internalising problem diagnosis (*Χ*^*2*^ = 0.48, *Bonferroni-corrected p* = 1.00).

**Table 4:** Comparison of the matched autism, ADHD and neurotypical control groups.

Variables	Autism (*n* = 73)	ADHD (*n* = 73)	Neurotypical (*n* = 146)	Group Comparisons ^a^			
	M (SD)	M (SD)	M (SD)	F	*p*	ω^2^ [95% CI] ^b^	BF_10_^c^
Age	35.40 (12.32)	33.64 (10.00)	34.77 (11.29)	0.50	0.609	0.00 [0.00,1.00]	0.06
Education	4.58 (2.05)	4.81 (2.03)	4.68 (1.98)	0.24	0.787	0.01 [0.00,1.00]	0.05

				*Χ* ^***2***^	*p*	*V* [95% CI] ^d^	*BF*_*10*_^c^
Sex (M: F)	44:29	44:29	90:56	0.06	0.972	0.01[0.00,0.09]	0.03

^a^ Robust (Welch) ANOVAs are reported for age and education.

^b^ Omega squared measure of effect size (0.01 = small, 0.06 = medium, 0.14 = large) with 95% confidence intervals shown in square brackets.

^c^ Bayes Factors quantifying the strength of the evidence for the null hypothesis (i.e., there is no group difference) compared to the alternative hypothesis (i.e., there is a group difference). For conventions on Bayes Factor interpretation, see ^50^.

^d^ Cramer’s *V* measure of effect size (at two degrees of freedom, 0.07 = small, 0.21 = medium, 0.35 = large) with 95% confidence intervals shown in square brackets.

The association between neurodevelopmental condition diagnosis and depression diagnosis was also significant: *Χ*^*2*^(2, *N* = 292) = 24.12, *p* < 0.001, *V* [95% CI] = 0.29 [0.18, 0.41]. Consistent with the foregoing analyses, adults with ADHD had higher odds of being diagnosed with depression (*OR* [95% CI] = 3.81 [2.05, 7.17], with the neurotypical control group set as the reference category) than autistic adults (*OR* [95% CI] = 3.41 [1.83, 6.43], with the neurotypical control group set as the reference category). However, the follow-up tests indicated that, while both clinical groups were significantly more likely to have a depression diagnosis than the neurotypical control group (autism: *Χ*^*2*^ = 15.88, *Bonferroni-corrected p* < 0.001; ADHD: *Χ*^*2*^ = 19.10, *Bonferroni-corrected p* < 0.001), the autism and ADHD groups did not significantly differ from one another (*Χ*^*2*^ = 0.11, *Bonferroni-corrected p* = 1.00).

There was also a significant association between neurodevelopmental condition diagnosis and GAD diagnosis: *Χ*^*2*^(2, *N* = 292) = 29.99, *p* < 0.001, *V* [95% CI] = 0.32 [0.22, 0.44]. Interestingly, the odds ratios calculated with the neurotypical control group as the reference category followed the opposite pattern to the previous two analyses: autistic adults had greater odds of being diagnosed with GAD (*OR* [95% CI] = 4.64 [2.53, 8.66]) than adults with ADHD (*OR* [95% CI] = 3.35 [1.82, 6.22]). However, the follow-up tests again showed that, while both clinical groups were significantly more likely to be diagnosed with GAD than the neurotypical control group (autism: *Χ*^*2*^ = 26.25, *Bonferroni-corrected p* < 0.001; ADHD: *Χ*^*2*^ = 15.94, *Bonferroni-corrected p* < 0.001), these two clinical groups did not significantly differ from one another (*Χ*^*2*^ = 0.99, *Bonferroni-corrected p* = 0.962).

### Associations between probable autism, probable ADHD and mental health diagnoses

Group comparisons, shown in Table 5, indicate that the probable autism, probable ADHD and neurotypical control groups were well-matched on age, sex and education level. Chi-square tests of independence were used to compare the odds of having a mental health diagnosis between the three groups. The first analysis revealed a significant association between neurodevelopmental condition screening group (i.e., probable autism versus probable ADHD versus neurotypical) and internalising problem diagnosis: *Χ*^*2*^(2, *N* = 1728) = 33.25, *p* < 0.001, *V* [95% CI] = 0.14 [0.10, 0.19]. Follow-up tests indicated that adults who met the screening threshold for ADHD were significantly more likely to be diagnosed with an internalising problem than both the neurotypical control group (*Χ*^*2*^ = 33.26, *Bonferroni-corrected p* < 0.001) and the probable autism group (*Χ*^*2*^ = 10.12, *Bonferroni-corrected p* = 0.004). In contrast, adults who met the screening threshold for autism were not significantly more likely to be diagnosed with an internalising problem than the neurotypical control group (*Χ*^*2*^ = 4.08, *Bonferroni-corrected p* = 0.130). We further found that adults in the probable ADHD group had twice the odds of having an internalising problem diagnosis than those in the neurotypical control group (*OR* [95% CI] = 2.04 [1.60, 2.61]), while the probable autism group had similar odds to the neurotypical control group (*OR* [95% CI] = 1.30 [1.01, 1.67]).

**Table 5:** Comparison of the matched probable autism, probable ADHD and neurotypical control groups.

Variables	Autism (*n* = 432)	ADHD (*n* = 432)	Neurotypical (*n* = 864)	Group Comparisons ^a^			
	M (SD)	M (SD)	M (SD)	F	*p*	ω^2^ [95% CI] ^b^	BF_10_^c^
Age	34.23 (11.13)	34.28 (11.37)	34.34 (11.10)	0.02	0.983	0.00 [0.00, 1.00]	0.01
Education	4.78 (1.87)	4.83 (1.85)	4.89 (1.86)	0.54	0.582	0.00 [0.00, 1.00]	0.01

				*Χ* ^***2***^	*p*	*V* [95% CI] ^d^	BF_*10*_^c^
Sex (M: F)	180:252	178:254	325:539	2.66	0.265	0.04 [0.00, 0.09]	0.02

^a^ Robust (Welch) ANOVAs are reported for age and education.

^b^ Omega squared measure of effect size (0.01 = small, 0.06 = medium, 0.14 = large) with 95% confidence intervals shown in square brackets.

^c^ Bayes Factors quantifying the strength of the evidence for the null hypothesis (i.e., there is no group difference) compared to the alternative hypothesis (i.e., there is a group difference). For conventions on Bayes Factor interpretation, see ^50^.

^d^ Cramer’s *V* measure of effect size (at two degrees of freedom, 0.07 = small, 0.21 = medium, 0.35 = large) with 95% confidence intervals shown in square brackets.

The association between neurodevelopmental condition screening group and GAD was also significant: *Χ*^*2*^(2, *N* = 1728) = 32.42, *p* < 0.001, *V* [95% CI] = 0.14 [0.09, 0.19]. Follow-up tests again indicated that the probable ADHD group was significantly more likely to be diagnosed with GAD than both the neurotypical control group (*Χ*^*2*^ = 32.51, *Bonferroni-corrected p* < 0.001) and the probable autism group (*Χ*^*2*^ = 7.72, *Bonferroni-corrected p* = 0.016). Once correcting for multiple comparisons, the probable autism group was not significantly more likely to be diagnosed with GAD than the neurotypical control group (*Χ*^*2*^ = 5.63, *Bonferroni-corrected p* = 0.053). Calculating odds ratios revealed that adults who met the screening threshold for ADHD had twice the odds of having a GAD diagnosis than neurotypical adults (*OR* [95% CI] = 2.18 [1.66, 2.86]), while those who met the screening threshold for autism had only slightly higher odds to neurotypical adults (*OR* [95% CI] = 1.42 [1.06, 1.89]).

Consistent with the foregoing analyses, the association between neurodevelopmental condition screening group and depression was significant: *Χ*^*2*^(2, *N* = 1728) = 22.38, *p* < 0.001, *V* [95% CI] = 0.11 [0.07, 0.16]. Once again, the follow-up tests indicated that the probable ADHD group was significantly more likely to be diagnosed with depression than both the neurotypical control group (*Χ*^*2*^ = 22.07, *Bonferroni-corrected p* < 0.001) and the probable autism group (*Χ*^*2*^ = 8.21, *Bonferroni-corrected p* = 0.013). As before, the probable autism group and neurotypical control group did not significantly differ in their associations with depression diagnosis (*Χ*^*2*^ = 1.64, *Bonferroni-corrected p* = 0.603). Compared to the neurotypical control group, the probable ADHD group had around twice the odds of having a depression diagnosis (*OR* [95% CI] = 1.92 [1.46, 2.53]) while the probable autism group had similar odds of having a depression diagnosis (*OR* [95% CI] = 1.21 [0.90, 1.63]).

## Discussion

We aimed to determine whether autism or ADHD is more strongly linked to clinically diagnosed internalising problems like anxiety and depression. Consistent with our previous findings^38^ and the wider literature (e.g.,^10,20^), both autism and ADHD were uniquely associated with an increased likelihood of having an internalising problem diagnosis. At the trait level, replicating our previous study^38^, these relationships were stronger for ADHD than autism, suggesting that an increase in the level of ADHD characteristics confers a greater risk for anxiety and depression than similar increases in autism characteristics. Interestingly, our case-control analyses, focusing on participants with clinically diagnosed autism and ADHD, revealed a more nuanced pattern of results. While participants with ADHD had greater odds of being diagnosed with both depression and overall internalising problems (i.e., GAD and/or depression) relative to neurotypical participants than did autistic participants, the reverse was true for GAD diagnosis. However, direct comparisons between the associations of autism and ADHD with these mental health diagnoses revealed non-significant differences. This suggests that both groups have similarly elevated odds of being diagnosed with internalising problems. That is, the differential links of autism and ADHD with internalising problems may be less pronounced at the clinical than trait level.

Differences in the pattern of findings between the trait-level and clinical analyses suggest that, while neurodevelopmental traits allow us to approximate the symptoms associated with such conditions, there may be qualitative differences between people who do and do not meet the diagnostic criteria. There even appears to be a difference between adults who meet the threshold for probable autism or ADHD and those who have clinical diagnoses, highlighting that screening measures cannot fully substitute diagnoses when investigating clinical samples. Indeed, our replication of the case-control analyses with groups formed using the AQ-Short and ASRS screeners showed that adults who met the screening threshold for ADHD were consistently the most likely to have mental health diagnoses. This poses an interesting challenge for studying the complex links between neurodivergence and mental health. Indeed, Abu-Akel and colleagues^51^ demonstrated that autistic traits are dimensionally distributed across the population, but this distribution also has a two-component structure that represents a categorical difference between the autistic and non-autistic individuals. A similar study with respect to ADHD traits and clinically diagnosed ADHD remains to be conducted in adults (though see^52,53^). Thus, it is possible that the psychological mechanisms that underpin the links between neurodevelopmental conditions and internalising problems at the clinical level may somewhat differ from those seen at the trait level in the wider population. This is a priority for future research, especially as we move towards a more dimensional conceptualisation of neurodivergence in line with the neurodiversity movement (see also^54^).

For now, we tentatively speculate that the consistently strong link between ADHD and internalising problems at the trait level may be driven by response inhibition difficulties (see^55–58^). However, within clinically diagnosed samples, a potentially stronger and clearer link between ADHD and depression may emerge through additional (albeit related) mechanisms, such as emotional dysregulation and irritability (see^59^). Indeed, both cross-sectional and longitudinal studies using youth samples (e.g., ^60–62^) have hinted that emotional dysregulation plays a mediating role in the relationship between ADHD and depression. Similarly, Bodalski and colleagues^63^ showed that ADHD was linked to internalising symptoms in adults through difficulties in modulating their emotional responses to stimuli.

In contrast, the strong association that we observed between autism and anxiety within our clinical sample may be underpinned by psychological processes like intolerance of uncertainty. Indeed, autistic individuals often demonstrate a strong preference for predictability and an increased sensitivity to uncertainty (e.g., ^64–66^). This dispositional trait is also commonly observed in people with anxiety^67–69^ and is a key target for psychological interventions^67,70,71^. Crucially, recent research has highlighted that intolerance of uncertainty might provide a framework for understanding the co-occurrence of autism and anxiety disorders in both children and adults^72–74^. Based on our findings, it may be prudent to measure and account for ADHD in such research, for a precise understanding of the interrelations between neurodevelopmental and mental health conditions.

Indeed, although the present study was not designed to test for the different psychological mechanisms that may underpin the relationships of autism and ADHD with internalising problems, we hope that our important correlational findings can serve as a springboard for such future cognitive and experimental mechanistic research. Our results also highlight the urgent clinical need to better understand these links to be able to offer effective mental health support to neurodivergent individuals. In addition to exploring the aforementioned psychological mechanisms, it is also important to consider potential environmental factors (e.g., physical surroundings, social network) that may contribute to the strong associations of autism and ADHD with mental health difficulties (see^8^). This line of investigation, which draws on the social model of disability^7,75^, could help to identify important environmental modifications that could be made to improve wellbeing in autism and ADHD.

Further research is also needed to address some of the limitations of our study. First, it will be crucial to understand how internalising problems may manifest in individuals who have concurrent autism and ADHD, as our sample did not have a sufficient number of participants with both diagnoses to test this. As it has only been possible to have a diagnosis of both autism and ADHD since 2013^1,31^, it will take some time before there are large enough samples to undertake this work in adulthood. Second, although we matched the three groups in our case-control analyses on age, sex, and education level, we did not consider other potentially confounding variables, such as participants’ ethnicity or their socioeconomic status. As research has indicated a potential link between these demographic factors and mental health (e.g., ^76–78^), it will be important to assess whether the magnitude of the associations between the different groups and poor mental health changes when these additional variables are considered. Finally, as the original data were collected online, we relied on participants’ self-reported traits and diagnostic status. While this allowed us to assess important relationships between neurodevelopmental and mental health conditions, our study would benefit from a replication in clinical populations with diagnoses from independent clinicians and health professionals (e.g., from community mental health and neurodevelopmental services), as well as looking at behavioural and cognitive features of autism and ADHD.

Overall, our findings demonstrate that, at both the trait and clinical level, autism and ADHD independently predict internalising problems like depression and GAD. In general, ADHD appears to be a stronger predictor of internalising problems, however, the pattern of associations between clinically diagnosed conditions is more nuanced. Specifically, adults with a diagnosis of ADHD are potentially more likely to experience depression than autistic adults but having an autism diagnosis may be more strongly associated with GAD than having an ADHD diagnosis. While further research is needed to replicate these findings and better understand the psychological mechanisms that underpin the observed relationships, our study offers important new evidence on the link between neurodevelopmental conditions and mental health in adulthood.

## Supplementary Information

Below is the link to the electronic supplementary material.

Supplementary Material 1

## Data Availability

The open access secondary data analysed in this study are available at: https://github.com/Lucy-Wal/Autism_ADHD_Adults.The analysis scripts and information about the R packages used for the analyses are available at: 10.17605/OSF.IO/DZVMG.
