# Supplementary material for: Neurodiversity and mental health in adulthood: exploring the unique contributions of autism and ADHD to internalising problems
Source: Sci Rep. 2026 Apr 1;16:16343. doi: 10.1038/s41598-026-35440-6 (PMC13212707; doi:10.1038/s41598-026-35440-6)
Supplement: Supplementary file 1 — Supplementary Material 1 [file 41598_2026_35440_MOESM1_ESM.docx]

**Supplementary Materials**

*Neurodiversity and mental health in adulthood: Exploring the unique contributions of autism and ADHD to internalising problems*

For the analysis scripts and information about the R packages used for the analyses, please see: <https://doi.org/10.17605/OSF.IO/DZVMG>

The open access secondary data analysed in this study are available at: <https://github.com/Lucy-Wal/Autism_ADHD_Adults>.

**STATISTICAL GROUP MATCHING**

**Case Control Analyses: Matching the Autism-only, ADHD-only and Neurotypical Control Groups**

Nearest neighbour matching^[1,2]^ was used to create an autism-only, an ADHD-only and a neurotypical control group for the case control analyses. We used the MatchIt package in R (version 4.7.1^[3]^) to match the groups as closely as possible in terms of age, sex and education level. To make the matching process reproducible, we set the random seed to 2024.

The two clinical groups were matched first. To ensure that we obtained distinct clinical groups, any participants who self-reported concurrent autism and ADHD diagnoses (*n* = 17) were removed from the data. Before commencing the matching process, Frequentist (Welch) and Bayesian t-tests were used to determine the extent to which the unmatched autism (*n* = 73) and ADHD (*n* = 87) groups differed on age and education level, and Frequentist and Bayesian chi-square tests of independence were used to compare the groups in terms of sex (see *Table S1*). The magnitude of the difference between the unmatched clinical groups determined the order in which the matching variables were entered into the matching algorithm. Specifically, age was entered first as it differed the most between the autism and ADHD groups, followed by education level and then sex. The two groups were compared again following the nearest neighbour matching process to ensure that the autistic (*n* = 73) and ADHD (*n* = 73) participants were adequately matched (see *Table S1*).

| **Table S1**  *Matching the Autism and ADHD Groups* | | | | | | |
| --- | --- | --- | --- | --- | --- | --- |
| *Pre-Matching Comparisons* | | | | | | |
| **Variables** | **Autism (*n* = 73)** | **ADHD (*n* = 87)** | **Group Comparisons ^a^** | | | |
|  | ***M (SD)*** | ***M (SD)*** | ***t*** | ***p*** | ***g* [95% CI] ^b^** | ***BF_10_* ^c^** |
| Age | 35.40 (12.32) | 32.30 (9.80) | -1.74 | 0.085 | -0.28 [-0.59, 0.03] | 0.72 |
| Education | 4.58 (2.05) | 5.03 (1.95) | 1.44 | 0.151 | 0.23 [-0.08, 0.54] | 0.45 |
|  |  |  | ***χ^2^*** | ***p*** | ***V* [95% CI] ^d^** | ***BF_10_* ^c^** |
| Sex (M:F) | 44:29 | 54:33 | 0.05 | 0.816 | 0.02 [0.00, 0.18] | 0.20 |
| *Post-Matching Comparisons* | | | | | | |
| **Variables** | **Autism (*n* = 73)** | **ADHD (*n* = 73)** | **Group Comparisons ^a^** | | | |
|  | ***M (SD)*** | ***M (SD)*** | ***t*** | ***p*** | ***g* [95% CI] ^b^** | ***BF_10_* ^c^** |
| Age | 35.40 (12.32) | 33.64 (10.00) | -0.94 | 0.347 | -0.16 [-0.48, 0.17] | 0.27 |
| Education | 4.58 (2.05) | 4.81 (2.03) | 0.69 | 0.491 | 0.11 [-0.21, 0.44] | 0.22 |
|  |  |  | ***χ^2^*** | ***p*** | ***V* [95% CI] ^d^** | ***BF_10_* ^c^** |
| Sex (M:F) | 44:29 | 44:29 | 0.00 | 1.00 | 0.00 [0.00, 1.00] | 0.20 |
| *Note.*  **^a^** Robust (Welch) t-tests are reported for age and education.  **^b^** Hedges’ *g* measure of effect size (0.20 = small, 0.50 = medium, 0.80 = large) with 95% confidence intervals shown in square brackets.  **^c^** Bayes Factors quantifying the strength of the evidence for the null hypothesis (i.e., there is no group difference) compared to the alternative hypothesis (i.e., there is a group difference). For conventions on Bayes Factor interpretation, see ^[4]^.  **^d^** Cramer’s *V* measure of effect size (at one degree of freedom, 0.10 = small, 0.30 = medium, 0.50 = large) with 95% confidence intervals shown in square brackets. | | | | | | |

Prior to the second round of nearest neighbour matching, the matched autism and ADHD groups were combined to form a ‘clinical’ group (*n* = 146). Frequentist (Welch) and Bayesian t-tests were then used to compare this clinical group with the unmatched neurotypical control group (*n* = 4819) on age and education level, and Frequentist and Bayesian chi-square tests of independence were used to compare the groups in terms of sex (see *Table S2*). As before, the magnitude of difference between the clinical and unmatched neurotypical control groups informed the order of variable entry in the matching algorithm, with age being entered first, followed by sex and finally education level. Following the nearest neighbour matching, the autism (*n* = 73), ADHD (*n* = 73) and neurotypical control (*n* = 146) groups were compared using Frequentist and Bayesian one-way ANOVAs (for age and education level) and Frequentist and Bayesian chi-square tests of independence (for sex). These analyses, presented in *Table 4* of the main article, demonstrated that the three groups were well-matched.

| **Table S2**  *A Pre-Matching Comparison of the Clinical and Neurotypical Control Groups* | | | | | | |
| --- | --- | --- | --- | --- | --- | --- |
| **Variables** | **Clinical (*n* = 146)** | **Neurotypical (*n* = 4819)** | **Group Comparisons ^a^** | | | |
|  | ***M (SD)*** | ***M (SD)*** | ***t*** | ***p*** | ***g* [95% CI] ^b^** | ***BF_10_* ^c^** |
| Age | 34.52 (11.21) | 39.37 (13.43) | 5.12 | < 0.001 | 0.36 [0.20, 0.53] | 838.76 |
| Education | 4.69 (2.03) | 4.87 (1.91) | 1.04 | 0.298 | 0.09 [-0.07, 0.26] | 0.17 |
|  |  |  | ***χ^2^*** | ***p*** | ***V* [95% CI] ^d^** | ***BF_10_* ^c^** |
| Sex (M:F) | 88:58 | 2371:2448 | 6.95 | 0.008 | 0.04 [0.02, 0.07] | 3.38 |
| *Note.*  **^a^** Robust (Welch) t-tests are reported for age and education.  **^b^** Hedges’ *g* measure of effect size (0.20 = small, 0.50 = medium, 0.80 = large) with 95% confidence intervals shown in square brackets.  **^c^** Bayes Factors quantifying the strength of the evidence for the null hypothesis (i.e., there is no group difference) compared to the alternative hypothesis (i.e., there is a group difference). For conventions on Bayes Factor interpretation, see ^[4]^.  **^d^** Cramer’s *V* measure of effect size (at one degree of freedom, 0.10 = small, 0.30 = medium, 0.50 = large) with 95% confidence intervals shown in square brackets. | | | | | | |

**Case Control Analyses: Matching the Probable Autism, Probable ADHD and Neurotypical Control Groups**

We used nearest neighbour matching^[1,2]^  to create an autism-only, an ADHD-only and a neurotypical control group based on the AQ-Short and ASRS screeners. We used the MatchIt package in R (version 4.7.1^[3]^) to match the groups as closely as possible on age, sex and education level. To make the matching process reproducible, we set the random seed to 2024.

The two clinical groups were matched first. To ensure that we obtained distinct clinical groups, any participants who met the screening threshold for both autism and ADHD (*n* = 902) were removed from the data. Before commencing the group matching process, we used Frequentist (Welch) and Bayesian t-tests to compare the unmatched probable autism (*n* = 1699) and probable ADHD (*n* = 432) groups on age and education level, and Frequentist and Bayesian chi-square tests of independence to compare them in terms of sex (see *Table S3*). The magnitude of the difference between the unmatched clinical groups determined the order in which the matching variables were entered into the matching algorithm. Specifically, age was entered first as it differed the most between the two groups, followed by sex and then education level. The groups were compared again following the nearest neighbour matching process to ensure that the probable autistic (*n* = 432) and probable ADHD (*n* = 432) participants were adequately matched (see *Table S3*).

| **Table S3**  *Matching the Autism and ADHD Groups based on the AQ-Short and ASRS Screeners* | | | | | | |
| --- | --- | --- | --- | --- | --- | --- |
| *Pre-Matching Comparisons* | | | | | | |
| **Variables** | **Autism (*n* = 1699)** | **ADHD (*n* = 432)** | **Group Comparisons ^a^** | | | |
|  | ***M (SD)*** | ***M (SD)*** | ***t*** | ***p*** | ***g* [95% CI] ^b^** | ***BF_10_* ^c^** |
| Age | 40.37 (13.38) | 34.28 (11.37) | 9.57 | < 0.001 | 0.47 [0.36, 0.57] | 5.85 x 10^14^ |
| Education | 4.83 (1.92) | 4.83 (1.85) | -0.03 | 0.973 | -0.00 [-0.11, 0.10] | 0.06 |
|  |  |  | ***χ^2^*** | ***p*** | ***V* [95% CI] ^d^** | ***BF_10_* ^c^** |
| Sex (M:F) | 938:761 | 178:254 | 27.08 | < 0.001 | 0.11 [0.07, 0.16] | 51293.62 |
| *Post-Matching Comparisons* | | | | | | |
| **Variables** | **Autism (*n* = 432)** | **ADHD (*n* = 432)** | **Group Comparisons ^a^** | | | |
|  | ***M (SD)*** | ***M (SD)*** | ***t*** | ***p*** | ***g* [95% CI] ^b^** | ***BF_10_* ^c^** |
| Age | 34.23 (11.13) | 34.28 (11.37) | -0.07 | 0.945 | -0.00 [-0.14, 0.13] | 0.08 |
| Education | 4.78 (1.87) | 4.83 (1.85) | -0.42 | 0.675 | -0.03 [-0.16, 0.10] | 0.08 |
|  |  |  | ***χ^2^*** | ***p*** | ***V* [95% CI] ^d^** | ***BF_10_* ^c^** |
| Sex (M:F) | 180:252 | 178:254 | 0.02 | 0.890 | 0.00 [0.00, 0.07] | 0.08 |
| *Note.*  **^a^** Robust (Welch) t-tests are reported for age and education.  **^b^** Hedges’ *g* measure of effect size (0.20 = small, 0.50 = medium, 0.80 = large) with 95% confidence intervals shown in square brackets.  **^c^** Bayes Factors quantifying the strength of the evidence for the null hypothesis (i.e., there is no group difference) compared to the alternative hypothesis (i.e., there is a group difference). For conventions on Bayes Factor interpretation, see ^[4]^.  **^d^** Cramer’s *V* measure of effect size (at one degree of freedom, 0.10 = small, 0.30 = medium, 0.50 = large) with 95% confidence intervals shown in square brackets. | | | | | | |

Before commencing the second round of nearest neighbour matching, the matched probable autism and probable ADHD groups were combined to form a ‘clinical’ group (*n* = 864). Frequentist (Welch) and Bayesian t-tests were then used to compare this clinical group with the unmatched neurotypical control group (*n* = 1963) on age and education level, and Frequentist and Bayesian chi-square tests of independence were used to compare the groups in terms of sex (see *Table S4*). Consistent with the first round of matching, the magnitude of difference between the clinical and unmatched neurotypical control groups informed the order of variable entry in the matching algorithm, with age being entered first, followed by sex and finally education level. After the nearest neighbour group matching, the probable autism (*n* = 432), probable ADHD (*n* = 432) and neurotypical control (*n* = 864) groups were compared using Frequentist and Bayesian one-way ANOVAs (for age and education level) and Frequentist and Bayesian chi-square tests of independence (for sex). These analyses, presented in *Table 5* of the main article, demonstrated that the three groups were well-matched.

| **Table S4**  *A Pre-Matching Comparison of the Clinical and Neurotypical Control Groups based on the AQ-Short and ASRS Screeners* | | | | | | |
| --- | --- | --- | --- | --- | --- | --- |
| **Variables** | **Clinical (*n* = 864)** | **Neurotypical (*n* = 1963)** | **Group Comparisons ^a^** | | | |
|  | ***M (SD)*** | ***M (SD)*** | ***t*** | ***p*** | ***g* [95% CI] ^b^** | ***BF_10_* ^c^** |
| Age | 34.25 (11.24) | 40.63 (14.06) | 12.83 | < 0.001 | 0.48 [0.40, 0.56] | 7.59 x 10^27^ |
| Education | 4.80 (1.86) | 4.97 (1.89) | 2.13 | 0.034 | 0.09 [0.01, 0.17] | 0.42 |
|  |  |  | ***χ^2^*** | ***p*** | ***V* [95% CI] ^d^** | ***BF_10_* ^c^** |
| Sex (M:F) | 358:506 | 927:1036 | 8.11 | 0.004 | 0.05 [0.03, 0.09] | 2.95 |
| *Note.*  **^a^** Robust (Welch) t-tests are reported for age and education.  **^b^** Hedges’ *g* measure of effect size (0.20 = small, 0.50 = medium, 0.80 = large) with 95% confidence intervals shown in square brackets.  **^c^** Bayes Factors quantifying the strength of the evidence for the null hypothesis (i.e., there is no group difference) compared to the alternative hypothesis (i.e., there is a group difference). For conventions on Bayes Factor interpretation, see ^[4]^.  **^d^** Cramer’s *V* measure of effect size (at one degree of freedom, 0.10 = small, 0.30 = medium, 0.50 = large) with 95% confidence intervals shown in square brackets. | | | | | | |

**REFERENCES**

1. Rubin, D. B. Matching to Remove Bias in Observational Studies. *Biometrics* **29**, 159–183 (1973).

2. Stuart, E. A. Matching methods for causal inference: A review and a look forward. *Stat Sci* **25**, 1–21 (2010).

3. Ho, D., Imai, K., King, G. & Stuart, E. A. MatchIt: Nonparametric Preprocessing for Parametric Causal Inference. *Journal of Statistical Software* **42**, 1–28 (2011).

4. Wagenmakers, E.-J., Wetzels, R., Borsboom, D. & van der Maas, H. L. J. Why psychologists must change the way they analyze their data: The case of psi: Comment on Bem (2011). *Journal of Personality and Social Psychology* **100**, 426–432 (2011).
